# CURE-SMOTE algorithm and hybrid algorithm for feature selection and parameter optimization based on random forests

**DOI:** 10.1186/s12859-017-1578-z

**Published:** 2017-03-14

**Authors:** Li Ma, Suohai Fan

**Affiliations:** School of Information Science and Technology, Jinan University, Guangzhou, 510632 China

**Keywords:** Random forests, Imbalance data, Intelligence algorithm, Feature selection, Parameter optimization

## Abstract

**Background:**

The random forests algorithm is a type of classifier with prominent universality, a wide application range, and robustness for avoiding overfitting. But there are still some drawbacks to random forests. Therefore, to improve the performance of random forests, this paper seeks to improve imbalanced data processing, feature selection and parameter optimization.

**Results:**

We propose the CURE-SMOTE algorithm for the imbalanced data classification problem. Experiments on imbalanced UCI data reveal that the combination of Clustering Using Representatives (CURE) enhances the original synthetic minority oversampling technique (SMOTE) algorithms effectively compared with the classification results on the original data using random sampling, Borderline-SMOTE1, safe-level SMOTE, C-SMOTE, and k-means-SMOTE. Additionally, the hybrid RF (random forests) algorithm has been proposed for feature selection and parameter optimization, which uses the minimum out of bag (OOB) data error as its objective function. Simulation results on binary and higher-dimensional data indicate that the proposed hybrid RF algorithms, hybrid genetic-random forests algorithm, hybrid particle swarm-random forests algorithm and hybrid fish swarm-random forests algorithm can achieve the minimum OOB error and show the best generalization ability.

**Conclusion:**

The training set produced from the proposed CURE-SMOTE algorithm is closer to the original data distribution because it contains minimal noise. Thus, better classification results are produced from this feasible and effective algorithm. Moreover, the hybrid algorithm's F-value, G-mean, AUC and OOB scores demonstrate that they surpass the performance of the original RF algorithm. Hence, this hybrid algorithm provides a new way to perform feature selection and parameter optimization.

## Background

Tin Kam Ho proposed the random forests (RF) concept [1] and the Random Subspace algorithm [2] in 1995 and 1998, respectively. Breiman [3] proposed a novel ensemble learning classification, random forests, by combining bagging ensemble learning and Tin Kam Ho’s concept in 2001. The feature of random forests that allows for avoiding over-fitting makes it suitable for use as a data dimension reduction method for processing data with missing values, noise and outliers. Although random forests have been applied to many other fields such as biological prediction [4], fault detection [5], and network attacks [6], studies seeking to improve the algorithm itself are lacking. The RF algorithm still has some shortcomings; for example, it performs poorly for classification on imbalanced data, fails to control the model during specific operations, and is sensitive to parameter adjustment and random data attempts. Usually, there are two ways to improve RF: increase the accuracy of each individual classifier or reduce the correlation between classifiers.

First, it is possible to increase the classification accuracy in minor class samples of RF for imbalanced training sets through data preprocessing. Several types of methods [7–10] based on both data and algorithms exist for imbalanced data. Chen [11] found that undersampling provides results closer to the original samples than does oversampling for large-scale data. A novel sampling approach [12] based on sub-modularity subset selection was employed to balance the data and select a more representative data subset for predicting local protein properties. Similarly, an algorithm combining RF and a Support Vector Machine (SVM) with stratified sampling [13] yielded a better performance than did other traditional algorithms for imbalanced-text categorization, including RF, SVM, SVM with undersampling and SVM with oversampling. A novel hybrid algorithm [14] using a radial basis function neural network (RBFNN) integrated with RF was proposed to improve the ability to classify the minor class of imbalanced datasets. In addition, imbalanced data for bioinformatics is a well-known problem and widely found in biomedical fields. Applying RF with SMOTE to the CHOM, CHOA and Vero (A) datasets [15] is considered a remarkable improvement that is helpful in the field of functional and structural proteomics as well as in drug discovery. Ali S [16] processed imbalanced breast cancer data using the CSL technique, which imposes a higher cost on misclassified examples and develops an effective Cost-Sensitive Classifier with a GentleBoost Ensemble (Can-CSC-GBE). The Mega-Trend-Diffusion (MTD) technique [17] was developed to obtain the best results on breast and colon cancer datasets by increasing the samples of the minority class when building the prediction model.

Second, it is possible to improve algorithm construction. Because the decision trees in the original algorithm have the same weights, a weighted RF was proposed that used different weights that affected the similarity [18] between trees, out-of-bag error [19], and so on. Weighted RF has been shown to be better than the original RF algorithm [20]. Ma [21] combined Adaboost with RF and adaptive weights to obtain a better performance. The weight of attributes reduces the similarity among trees and improves RF [22]. Moreover, the nearest K-neighbour [23] and pruning mechanism can help achieve a better result when using margin as the evaluation criterion [24].

In this paper, the main work is divided into two parts: first, the CURE-SMOTE algorithm is combined with RF to solve the shortcomings of using SMOTE alone. Compared with results on the original data, random oversampling, SMOTE, Borderline SMOTE1, safe-level-SMOTE, C-SMOTE, and the k-means-SMOTE algorithm, CURE-SMOTE's effectiveness when classifying imbalanced data is verified. Then, to simultaneously optimize feature selection, tree size, and the number of sub-features, we propose a hybrid algorithm that includes a genetic-random forests algorithm (GA-RF), a particle swarm-random forests algorithm (PSO-RF) and an artificial fish swarm-random forests algorithm (AFSA-RF). Simulation experiments show that the hybrid algorithm obtains better features, selects better parameter values and achieves a higher performance than traditional methods.

## Methods

### Random forests algorithm review

#### Algorithm principle

**Fig. 1:**
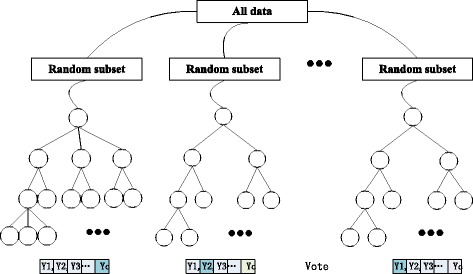
Random forests algorithm

RF is a combination of Bagging and Random Subspace, consisting of many binary or multi-way decision trees *h*_1_(*x*), *h*_2_(*x*), … *h*_*nTree*_(*x*), as shown in Fig. 1. The final decision is made by majority voting to aggregate the predictions of all the decision trees. The original dataset *T* = {(*x*_*i*1_, *x*_*i*2_, …, *x*_*iM*_, *y*_*i*_)}_*i* = 1_^*N*^ contains *N* samples, the vector *x*_*i*1_, *x*_*i*2_, …, *x*_*iM*_ denotes the *M*-dimension attributes or features, *Y* = {*y*_*i*_}_*i*_^*N*^ denotes classification labels, and a sample is deduced as label *c* by *y*_*i*_ = *c*.

**Fig. 2:**
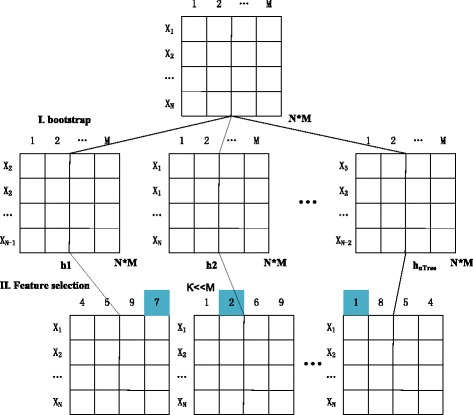
Two random procedures diagram

There are two random procedures in RF. First, training sets are constructed by using a bootstrap [25, 26] mechanism randomly with replacement [Fig. 2 (I)]. Second, random features are selected with non-replacement from the total features when the nodes of the trees are split. The size *κ* of the feature subset is usually far less than the size of the total features, *M*. The first step is to select *κ* features randomly, calculate the information gain of *κ* split and select the best features. Thus, the size of candidate features becomes *M* − *κ*_._ Then, continue as shown in Fig. 2 (II).

#### Classification rules and algorithmic procedure

The best attribute can be computed by three methods: information gain, information gain rate and Gini coefficient, which correspond to ID3, C4.5 [27] and CART [28], respectively. When the attribute value is continuous, the best split point must be selected. We use the CART method in this paper; hence, a smaller Gini coefficient indicates a better classification result. Let *P*_*i*_ represent the proportion of sample *i* in the total sample size. Assume that sample *T* is divided into *k* parts after splitting by attribute *A*.

1 $$ Gini(T)=1-{\displaystyle \sum_i^c{P}_i^2} $$

2 $$ Gini\left( T, A\right)={\displaystyle \sum_{j=1}^k\frac{\left|{T}_j\right|}{\left| T\right|} Gini\left({T}_j\right)} $$

There are several ways by which the termination criteria for RF can be met. For example, termination occurs when the decision tree reaches maximum depth, the impurity of the end node reaches the threshold, the number of final samples reaches a set point, and the candidate attribute is used up. The RF classification algorithm procedure is shown in Algorithm 1.
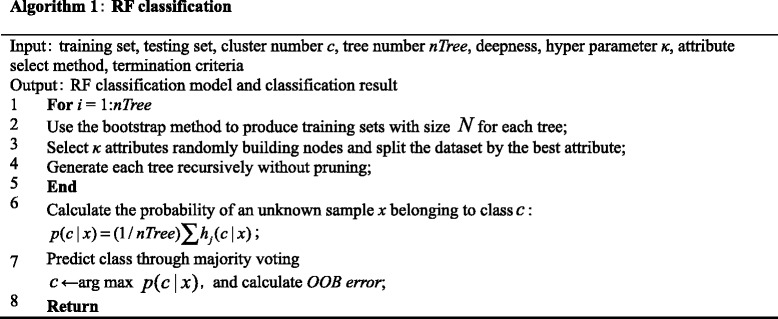


### CURE-SMOTE algorithm

#### Definition and impact of imbalanced data

In recent years, the problem of classifying imbalanced data [29] has attracted increasing attention. Imbalanced data sets generally refer to data that is distributed unevenly among different categories where the data in the smaller category is far less prevalent than data in the larger category. The Imbalance Ratio (IR) is defined as the ratio of the number of minor class samples to the number of major class samples. Therefore, imbalanced data causes the training set for each decision tree to be imbalanced during the first “random” procedure. The classification performance of traditional RF on imbalanced data sets [30] is even worse than that of SVMs [31].

#### SMOTE algorithm

Several methods exist for processing imbalanced data, including sample-based and algorithmic techniques, the combination of sampling and algorithm techniques, and feature selection. In particular, a type of synthesis resampling technique algorithm called the synthetic minority oversampling technique (SMOTE) [32–34], has a positive effect on the imbalanced data problem. The specific idea is implemented as follows: obtain the *k* -nearest neighbours of sample *X* in the minor class, select *n* samples randomly and record them as *X*_*i*_. Finally, the new sample *X*_*new*_ is defined by interpolation as follows:

3 $$ {X}_{new}={X}_{origin}+ rand\times \left({X}_i-{X}_{origin}\right), i=1,2,\dots, n, $$

where *rand* is a random number uniformly distributed within the range (0,1), and the ratio for generating new samples approximates [1/*IR*] − 1.

However, some flaws exist in the SMOTE algorithm. First, the selection of a value for *k* is not informed by the nearest neighbours selection. Second, it is impossible to completely reflect the distribution of original data because the artificial samples generated by the minor class samples at the edges may lead to problems such as repeatability and noisy, fuzzy boundaries between the positive and negative classes.

Therefore, researchers have sought to improve the SMOTE algorithm. The Borderline–SMOTE1 algorithm [35] causes new samples to be more effective using interpolation along the border areas, but it fails to find all the boundary points. Definitions for this algorithm are shown in Table 1: *m* is the number of nearest-neighbour samples in the minor class, and *k* is the number of samples in the major class.

**Table 1:** Definitions in Borderline-SMOTE 1

Point	Definition
Noisy point	*m* = *k*
Boundary point/dangerous point	*m*/2 ≤ *k* < *m*
Safe point	0 ≤ *k* < *m*/2

Motivated by Borderline–SMOTE 1, safe-level-SMOTE [36] advocates calculating the safe level of minor class samples, but it can easily fall into overfitting. Cluster-SMOTE [37] obtains a satisfactory classification effect for imbalanced datasets by using K-means to find clusters of minor class samples and then applying SMOTE. In addition, spatial structures have been studied such as N-SMOTE [38] and nuclear SMOTE [39]. The authors of [40] proposed an interpolation algorithm based on cluster centres. SMOTE was combined with a fuzzy nearest-neighbour algorithm in [41]. In [42], a preferable classification effect promoted by hierarchical clustering sampling was shown. Recently, a SMOTE noise-filtering algorithm [43] and MDO algorithms with Markov distance [44] have been proposed. In general, many improved versions of the SMOTE algorithm have been proposed, but none of these improvements seem perfect. This paper seeks to solve the shortcomings of SMOTE.

The K-means algorithm is effective only for spherical datasets and its application requires a certain amount of time. The CURE [45] hierarchical clustering algorithm is efficient for large datasets and suitable datasets of any shape dataset. Moreover, it is not sensitive to outlier and can recognize abnormal points. Consequently, CURE is better than the BIRCH, CLARANS and DBSCAN algorithms [46]. In the CURE algorithm, each sample point is assumed to be a cluster. These points are merged using local clustering until the end of the algorithm. Thus, the CURE algorithm is appropriate for distributed extensions. In this paper, inspired by C-SMOTE [40] and the hierarchical clustering sampling adaptive semi-unsupervised weighted oversampling (A-SUWO) [42] algorithms, the novel CURE-SMOTE algorithm is proposed to accommodate a wider range of application scenarios.

#### Design and analysis of CURE-SMOTE

The general idea of the CURE-SMOTE algorithm is as follows: cluster the samples of the minor class using CURE, remove the noise and outliers from the original samples, and, then, generate artificial samples randomly between representative points and the centre point. The implementation steps of the CURE-SMOTE algorithm are as follows:

- **Step 1**. Normalize the dataset, extract the minor class samples, *X*, and calculate the distance *dist* among them. Each point is initially considered as a cluster. For each cluster *U*, *Ur* and *Uc* represent the representative set and the centre point, respectively. For two data items *p* and *q*, the distance between the two clusters *U* and *V* is:
  4 $$ dist\left( U, V\right)=\underset{p\in Ur, q\in Vr}{ \min } dist\left( p, q\right). $$
- **Step 2**. Set the clustering number, *c*, and update the centre and representative points after clustering and merging based on the smallest distance of the two clusters,

5 $$ U c\leftarrow \frac{\left| U\right|\cdot U c+\left| V\right|\cdot Vc}{\left| U\left|+\right| V\right|} $$

6 $$ U r\leftarrow \left\{ p+\alpha \cdot \left( Uc- p\right)\Big| p\in U r\right\}, $$

where |*U*| is the number of data items for class *U*, and the shrinkage factor *α* is generally 0.5. The class with slowest growth speed is judged to contain abnormal points and will be deleted. If the number of representative points is larger than required, select the data point farthest from the clustering centre as the first representative point. Then, the next representative point is the one farthest from the former. When the number of clustering centres reaches a predefined setting, the algorithm terminates, and clusters containing only a few samples are removed.

- **Step 3**. Generate a new sample according to the interpolation formula. $\overline{X}$ represents the samples after clustering by the CURE algorithm.
  7 $$ {X}_{n ew}^n=\overline{X}+ rand\left(0,1\right)\times \left( Ur-\overline{X}\right). $$
- **Step 4**. Calculate *IR*, and return to Step 3 if *IR* ≤ *IR*_0_.
- **Step 5**. Finally, classify the new dataset as ${X}_{n ew}=\overline{X}\cup \left\{{X}_{n ew}^n\right\}$ and add samples of the major class by RF. The distance is measured using Euclidean distance. For example, the distance between sample *X*_1_ = (*X*_11_, *X*_12_ …, *X*_1*M*_) and sample *X*_2_ = (*X*_21_, *X*_22_ …, *X*_2*M*_) is ${d}_{12}=\sqrt{{\displaystyle \sum_{j=1}^M{\left({X}_{1 j}-{X}_{2 j}\right)}^2}}$.

During the clustering process of the CURE-SMOTE algorithm, noisy points must be removed because they are far away from the normal points, and they hinder the merge speed in the corresponding class. When clustering is complete, the clusters containing only a few samples are also deemed to be noisy points. For the sample points after clustering, the interpolation can effectively prevent generalization and preserve the original distribution attributes of the data set. In the interpolation formula, *X*_*i*_ is replaced by the representative points; consequently, the samples are generated only between the representative samples and the samples in the original minor class, which effectively avoids the influence of boundary points. The combination of the clustering and merge operations serves to eliminate the noise points at the end of the process and reduce the complexity because there is no need to eliminate the farthest generated artificial samples after the SMOTE algorithm runs. Moreover, all the termination criteria such as reaching the pre-set number of clusters, the number of representative samples, or the distance threshold, avoid setting the *k* value of the original SMOTE algorithm and, thus, reduce the instability of the proposed algorithm.

#### Research concerning feature selection and parameter optimization

Classification [47] and feature selection [48–50] are widely applied in bioinformatics applications such as gene selection [51, 52] and gene expression [53–55]. Chinnaswamy A [56] proposed a hybrid feature selection using correlation coefficients and particle swarm optimization on microarray gene expression data. The goal of feature selection is to choose a feature subset that retains most of the information of the original dataset, especially for high-dimensional data [57]. The authors of [58] showed that machine-learning algorithms achieve better results after feature selection. Kausar N. [59] proposed a scheme-based RF in which useful features were extracted from both the spatial and transform domains for medical image fusion. During the second "random" time of RF, a number of attributes were selected randomly to reduce the correlation between trees, but this operation promotes redundant features that may affect the generalization ability to some degree. Thus, new types of evaluation mechanisms were proposed based on the importance of the attributes [21, 60, 61], using weighted features as well as cost-sensitivity features [62], and so on; however, their calculations are comparatively complicated. Recently, researchers have combined the RF algorithm with intelligent algorithms. Such combinations have achieved good results in a variety of fields. In [5], an improved feature selection method based on GA and RF was proposed for fault detection that significantly reduces the OOB error. The results of [4, 6] indicate that a type of hybrid PSO-RF feature selection algorithm is widely applied in certain fields. However, the works mentioned above do not involve parameter optimization.

Three main parameters influence the efficiency and performance of RF: *nTree*—the size of the tree, *MinLeaf*—the minimum sample number of leaf nodes, and *κ* —the attribute subset size. Previous studies have shown that the classification performance of RF is less sensitive to *MinLeaf* [63]. A larger *nTree* increases the number of trees in the classifier, helps ensure the diversity of individual classifiers and, thus, improves performance. However, a larger *nTree* also increases the time cost and may lead to less interpretable results, while a small *nTree* results in increased classification errors and poor performance. Usually, *κ* is far less than the number of total attributes [64]. When all the similar attributes are used for splitting the tree nodes in the Bagging algorithm, the effect of the tree model worsens due to the higher similarity degree among trees [65]; when *κ* is smaller, the stronger effects of randomness lower the classification accuracy. The hyper parameter *κ* behaves differently for different issues [66]; hence, an appropriate value can cause the algorithm to have excellent performance for a specific problem. Breiman pointed out that selecting the proper *κ* value has a great influence on the performance of the algorithm [3] and suggested that the value should be 1, $\sqrt{M}$, $\frac{1}{2}\sqrt{M}$, $2\sqrt{M}$ and ⌊ log_2_(*M*) + 1⌋. Generally, *κ* is fixed as $\left\lfloor \sqrt{M}\right\rfloor$, but that value does not guarantee obtaining the best classifier. Therefore, the authors of [67] suggested that the minimum OOB error be used to obtain the approximate value to overcome the shortcomings of the orthogonal validation method. Moreover, OOB data has been used to estimate the optimal training sample proportion to construct the Bagging classifier [68]. To sum up, it is difficult for traditional parameter values to achieve an optimal performance. In terms of the search for the optimal parameter, typical approaches have incorporated exhaustive search, grid search, and orthogonal selection, but these methods have a high time complexity.

#### Review of intelligent algorithms

Because intelligent algorithms are superior for solving NP-hard problems and for optimizing parameters, they have been the subject of many relevant and successful studies [69–72].

The main idea behind the genetic algorithm (GA) is to encode unknown variables into chromosomes and change the objective function into fitness functions. The fitness value drives the main operations—selection, crossover and mutation—to search for the best potential individuals iteratively. Eventually the algorithm converges, and the optimal or a suboptimal solution of the problem is obtained. GA has the advantage of searching in parallel, and it is suitable for a variety of complex scenarios.

The particle swarm optimization (PSO) algorithm is theoretically simpler and more efficient than the GA [73]. The main idea behind PSO is to simulate the predation behaviour of birds. Each particle represents a candidate solution and has a position, speed and a fitness value. Historical information on the optimal solution instructs the particle to fly toward a better position.

The artificial fish swarm algorithm (AFSA) [74] is a novel algorithm with high potential. The main idea behind AFSA is to imitate the way that fish prey, swarm, follow and adopt random behaviours. The candidate solution is translated into the individual positions of the fish, while the objective function is converted to food concentration.

**Fig. 3:**
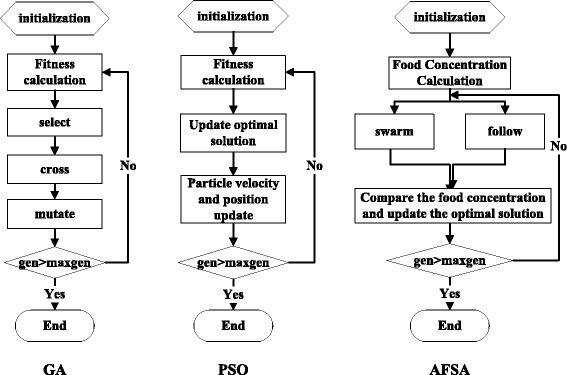
Diagrams of GA, PSO, and AFSA

Diagrams for GA, PSO and AFSA are shown in Fig. 3.

There is little research on optimizing the hyper parameter *κ* of random forests. In [67], the size of the decision tree is fixed at 500, but this approach achieves the optimal parameter on only half the dataset. Worse, it requires considerable time and is suitable for single parameter optimization only. This paper proposes combining a new hybrid algorithm for feature selection and parameter optimization with RF is proposed based on [4–6].

#### The proposed hybrid algorithm for feature selection and parameter optimization

We propose the hybrid GA-RF, PSO-RF or AFSA-RF algorithm for feature selection, parameter optimization and classification. The algorithm seeks to remove redundant features and attain the optimal feature subset and, finally, to explore the relation between performance and *nTree*, as well as the hyper parameter *κ*.

Generally, *p* -fold cross validation is used to traverse the parameter and to estimate the algorithm in the experiment, but time complexity is high. In this paper, OOB error replaces the cross-validation algorithm for binary classification, while the full misclassification error is used for multi-classification. Hence, the time complexity is reduced to 1/*p*. During the process, cross validation is required for classification.

Objective function:

8 $$ f\left( nTre{e}^{*},{\kappa}^{*},\left\{ Attribut{e}_i\Big| i=1,2\dots, M\right\}\right)= \arg \min \left(\operatorname{avg} OOB\  error\right) $$

Studies have shown that the larger *nTree* is, the more stable the classification accuracy will be. We set *nTree* and *κ* in the range [0, 500] and [1, M], respectively, by considering both the time and space complexities.

Optimization variables: *n*Tree, *κ*, {*Attribute*_*i*_|*i* = 1, 2 …, *M*}

Binary encoding involves two tangent points and three steps. Let *nTree* and *κ* be numbers in the binary system. A value of 0 in {*Attribute*_*i*_|*i* = 1, 2 …, *M*} represents an unselected feature in the corresponding position, while a 1 represents the selected features. The constraint condition is $\kappa \le {\displaystyle \sum_{i=1}^M Attribut{e}_i}$.

**Fig. 4:**
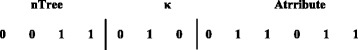
Binary coding

Then, an *nTree* is generated randomly between [0, 500]. Because 2^9^ = 512, a 9-bit length ensures a full set of variables. The bits used for *κ* and the bits used for the attributes are different for different data sets. The bits of *κ* are the binary representation of *M*, while the number of bits of the attributes are *M* (Fig. 4)_._ The initialization continues until a valid variable is generated.

**Fig. 5:**
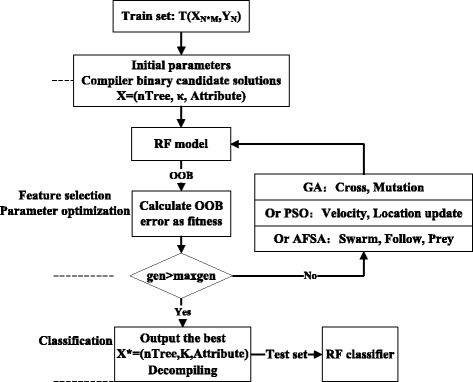
The diagram of a hybrid algorithm based on RF and an artificial algorithm

The diagram for a hybrid algorithm based on RF and an artificial algorithm for feature selection and parameter optimization is shown in Fig. 5.

#### Hybrid GA-RF

**Fig. 6:**
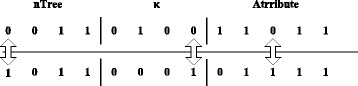
Crossover operation
- **Step 5**. Mutation operation: select an individual and a position *j* randomly to mutate by switching 0 and 1. When a feasible solution is achieved, calculate the fitness value and update the optimal solution. The mutation operation is shown in Fig. 7

- **Step 1**. Initialize the population: Perform binary encoding. The population size is set to *popsize*, the max iteration time is set to *maxgen*, the crossover probability is *P*_*c*_, and the mutation probability is *P*_*m*_.
- **Step 2**. Combine the GA with RF classification and calculate the fitness function, *F* = max(1/*f*), gen = 1.
- **Step 3**. Perform the selection operation with the roulette method: the probability of selecting an individual is dependent on the proportion of the overall fitness value that the individual represents:
  9 $$ {p}_i={F}_i/{\displaystyle \sum_{i=1}^{p opsize}{F}_i}. $$
- **Step 4**. Conduct the crossover operation with the single-point method: two selected individuals cross at a random position with different values. The offspring generation will be regenerated until it turns out to be legal. The process is shown in Fig. 6.

**Fig. 7:**
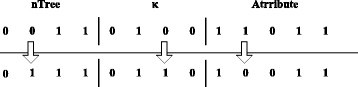
Mutation operation
- **Step 6**. When gen > maxgen, the algorithm will terminate; otherwise, return to Step 3.

#### Hybrid PSO-RF

- **Step 1**. Initialize the population. The population size is set to *popsize*, the max iteration time is set to *maxgen*, the position of the binary particle is *X*_*k*_ = {*Z*_*k*,1_, *Z*_*k*,2_, …}, *k* = 1, 2, … *popsize*, the velocity is *V*, the learning factors are *c*_1_, *c*_2_, and the weight is *w*.
- **Step 2**. Combine the PSO with RF classification and calculate the fitness function *F* = max(1/*f*), gen = 1.
- **Step 3**. Update the velocities *V* ^*k* + 1^ and positions *X* ^*k* + 1^ of particles. Let *P* ^*k*^ be the optimal position of an individual particle, *Pg* ^*k*^ be the optimal position of all particles, and *rand* be a random number uniformly distributed in the range (0,1):
  10 $$ {V}^{k+1}= w{V}^k+{c}_1{r}_1\left({P}^k-{X}^k\right)+{c}_2{r}_2\left( P{g}^k-{X}^k\right),{r}_1,{r}_2\in \left[0,1\right] $$
  11 $$ sigmoid\left({V}^{k+1}\right)=\frac{1}{1+{e}^{-{V}^{k+1}}} $$
  12 $$ {Z}_{k+1, j}=\left\{\begin{array}{c}\hfill 0,\hfill \\ {}\hfill 1,\hfill \end{array}\right.\kern2em \begin{array}{c}\hfill rand> sigmoid\left({V}^{k+1}\right)\hfill \\ {}\hfill rand\le sigmoid\left({V}^{k+1}\right)\hfill \end{array}\kern2em  rand\sim U\left(0,1\right). $$
- **Step 4**. If gen > maxgen, the algorithm will terminate; otherwise, return to Step 3.

#### Hybrid AFSA-RF

- **Step 1**. Initialize the population. The population size is set to *popsize*, the maximum number of iterations is set to *maxgen*, the fish positions are *X*_*k*_ = {*Z*_*k*,1_, *Z*_*k*,2_, …}, *k* = 1, 2, … *popsize*, the visual distance is *visual*, the crowding degree factor is *delta*, and the maximum number of behaviours to try is *try_number*.
- **Step 2**. Combine with RF classification and calculate the food concentration *F* = max(1/*f*);
- **Step 3**. Swarm and follow at the same time.
  - Swarm behaviour: The current state of a fish is *X*_*i*_, the number of partners in view is *nf*, and the centre position is *X*_*c*_. When $\frac{F_c}{nf}> delta\cdot Fitnes{s}_i$, move to the centre position according to the following formula; otherwise, conduct the prey behaviour.
    13 $$ {Z}_{k+1, i}=\left\{\begin{array}{l}\begin{array}{cc}\hfill {Z}_{k, i}\hfill & \hfill {Z}_{k, i}={Z}_{c, i}\hfill \end{array}\\ {}\begin{array}{cc}\hfill 0\hfill & \hfill {Z}_{k, i}\ne {Z}_{c, i}, rand>0.5\hfill \end{array}\\ {}\begin{array}{cc}\hfill 1\hfill & \hfill {Z}_{k, i}\ne {Z}_{c, i}, rand\le 0.5\hfill \end{array}\end{array}\right.. $$
  - Follow behaviour: Find the fish *X*_max_ with the maximum food concentration value, *F*_max_. If $\frac{F_{\max }}{nf}> delta\cdot {F}_i$, move to *X*_max_ and calculate the food concentration value. Then, update the food concentration value by comparing it with the value of the swarm behaviour; otherwise, conduct the prey behaviour.
    14 $$ {Z}_{k+1, i}=\left\{\begin{array}{l}\begin{array}{cc}\hfill {Z}_{k, i}\hfill & \hfill {Z}_{k, i}={Z}_{\max, i}\hfill \end{array}\\ {}\begin{array}{cc}\hfill 0\hfill & \hfill {Z}_{k, i}\ne {Z}_{\max, i}, rand>0.5\hfill \end{array}\\ {}\begin{array}{cc}\hfill 1\hfill & \hfill {Z}_{k, i}\ne {Z}_{\max, i}, rand\le 0.5\hfill \end{array}\end{array}\right.. $$
  - Prey behaviour: The current state is *X*_*k*_ = {*Z*_*k*,*i*_}, and the random selection state is *X*_*j*_ = {*Z*_*j*,*i*_} around the vision range with *d*_*ij*_ = *visual*_._ When *F*_*k*_ > *F*_*j*_,restart to generate the next state, *X*_*k* + 1_, and calculate the food concentration until *try_number* is reached; otherwise, terminate the prey behaviour according to the following function:
    15 $$ {Z}_{k+1, i}=\left\{\begin{array}{l}\begin{array}{cc}\hfill {Z}_{k, i}\hfill & \hfill {Z}_{k, i}={Z}_{j, i}\hfill \end{array}\\ {}\begin{array}{cc}\hfill 0\hfill & \hfill {Z}_{k, i}\ne {Z}_{j, i}, rand>0.5\hfill \end{array}\\ {}\begin{array}{cc}\hfill 1\hfill & \hfill {Z}_{k, i}\ne {Z}_{j, i}, rand\le 0.5\hfill \end{array}\end{array}\right.. $$
- **Step 4**. Update the state of the optimal fish. When gen > maxgen, the algorithm will terminate; otherwise, return to Step 3.

## Results and discussion

The experiments in this paper are divided into two parts. Experiment 1 explores the validity of the CURE-SMOTE algorithm. Experiment 2 investigates the effectiveness of the hybrid algorithm.

### Performance evaluation criteria

Referring to the evaluation used in [75], the measures of the quality of binary classification are built using a confusion matrix, where TP and FN are the numbers of correctly and incorrectly classified compounds of the actual positive class, respectively. Similarly, TN and FP denote the numbers of correctly and incorrectly classified compounds of the actual negative class.

The measures accuracy, sensitivity, specificity and precision are defined as follows.

16 $$ Accurcacy=\left( TP+ TN\right)/\left( TP+ TN+ FP+ FN\right)=\left( TP+ TN\right)/ N $$

17 $$ Sensitivity\ \mathrm{or}\  Recall= T P/\left( TP+ FN\right) $$

18 $$ Specificity= T N/\left( FP+ TN\right) $$

19 $$ Precision= T P/\left( TP+ FP\right) $$

The classifiers may have a high overall accuracy with 100% accuracy in the majority class while achieving only a 0–10% accuracy in the minority class because the overall accuracy is biased towards the majority class. Hence, the accuracy measure is not a proper evaluation metric for the imbalanced class problem. Instead, we suggest using F-value, Geometric Mean (G-mean) and AUC for imbalanced data evaluations.

The F-value measure is defined following [26]. A larger F-value indicates a better classifier. F-value is a performance metric that links both precision and recall:

20 $$ F=\frac{2}{1/ Precision+1/ Recall}. $$

The G-mean [76] attempts to maximize the accuracy across the two classes with a good balance and is defined as follows. Only when both sensitivity and specificity are high can the G-mean attain its maximum, which indicates a better classifier:

21 $$ G- mean=\sqrt{Sensitivity\cdotp Specificity}. $$

AUC is the area under the receiver operating characteristics (ROC) curve. AUC has been shown to be a reliable performance measure for imbalanced and cost-sensitive problems. An AUC–based permutation variable is presented in [77]; this approach is more efficient than the approach based on the OOB error.

The training set is obtained by using the bootstrap method. Because of repeated extraction, it contains only 63% of the original data; the 37% of the data that never appear are called "out-of- -bag" (OOB) data [78]. OOB estimation is an unbiased estimate of the RF algorithm and can be used to measure the classifier's generalization ability. A smaller OOB error indicates a better classification performance. OOB error is defined as follows:

22 $$ O O B\  erro r={\displaystyle \sum_i^{nTree} O O B\  erro{r}_i}/ nTree. $$

Margin is a new evaluation criterion that has been applied to the classification of remote sensing data [79]. The larger the margin is, the higher the classifier's credibility is:

23 $$ margin={\displaystyle \sum_i^{n Tree} margi{n}_i}/ nTree. $$

### Experiment 1 and parameter settings

The experiments were implemented using Matlab 2012a on a workstation with a 64-bit operating system, 2 GB of RAM and a 2.53 GHz CPU. Artificial Data Circle and UCI imbalanced datasets were selected for the experiments. More detailed information about five datasets is listed in Table 2. To simulate the actual situation appropriately and preserve the degree of imbalance of the original data, the training set and testing set were divided using stratified random sampling at a ratio of 3:1, except for SPECT. The SPECT.test dataset incorporates 187 samples, and the proportions of the classes labelled 1 and 0 are 84:103, respectively. The tree size is 100 and the depth is 20.

**Table 2:** Dataset

Id	Dataset	N	M	Positive class	Negative class	IR	Label
1	Circle	1362	2	229	1133	0.2021:1	1:0
2	Blood-transfusion	748	4	178	570	0.3123:1	4:2
3	Haberman's survival	306	3	81	225	0.36:1	2:1
4	Breast-cancer-wisconsin	702	10	243	459	0.5249:1	1:0
5	SPECT.train	80	23	26	54	0.4815	1:0

To verify the effectiveness of the CURE-SMOTE algorithm it was compared with the original data, random oversampling, SMOTE, Borderline-SMOTE1, safe-level SMOTE, C-SMOTE (using mean value as the centre) and k-means-SMOTE (shown in Table 3) algorithms. To evaluate the performance of the different algorithms, F-value, G-mean, AUC and OOB error are used as performance measures. The results of each experiment were averaged over 100 runs to eliminate random effects.

**Table 3:** Comparison of algorithms and references

Algorithm	Reference	Algorithm	Reference
SMOTE	[32]	Safe-level SMOTE	[36]
Borderline-SMOTE 1	[35]	C-SMOTE	[36]
k-means-SMOTE	[37]	-	-

**Fig. 8:**
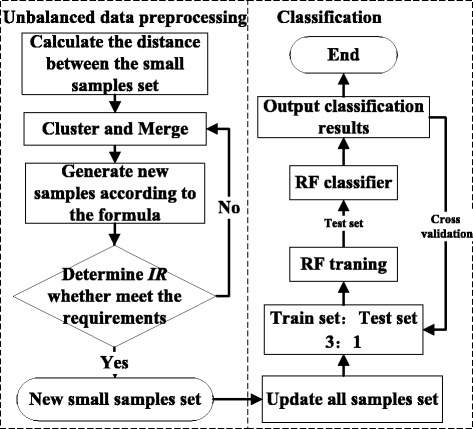
CURE-SMOTE algorithm diagram

To facilitate the comparisons, *m* and *k* were set to 20 and 5, respectively, in SMOTE, Borderline-SMOTE1 and safe-level-SMOTE. The number of clusters in C-SMOTE and k-means-SMOTE were set to five. Following the suggested setting for the CURE algorithm, the cluster results are better when the constriction factor is in the range [0.2, 0.7] and when the number of representative points is greater than 10. Thus, the constriction factor was set to 0.5 and the number of representative points was set to 15. The number of clusters was set to two in the circle, while the others were all five. Samples were removed when the number of representative points did not increase for ten iterations or when the sample size of the cluster class was less than 1/(10*c*) of the total sample size when clustering was complete. In the experiments in this paper, *IR*_0_ was fixed at 0.7. The CURE-SMOTE algorithm diagram is depicted in Fig. 8.

### Results and discussion of CURE - SMOTE algorithm

**Fig. 9:**
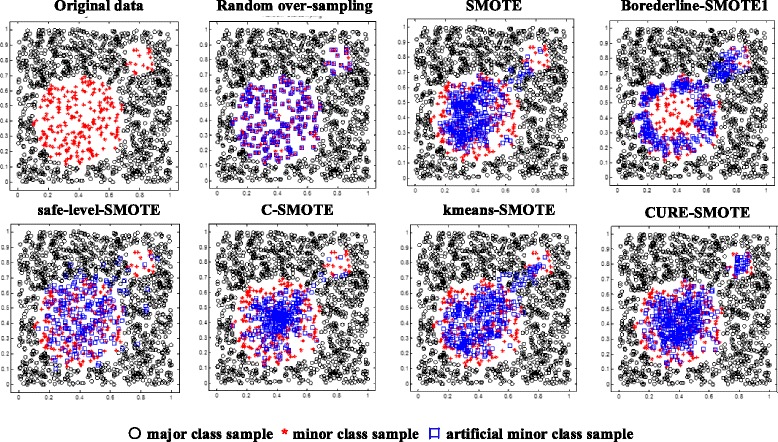
Artificial samples generated by different methods

**Fig. 10:**
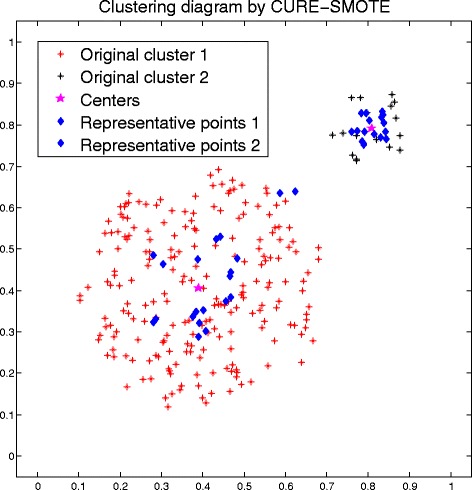
The CURE clustering result

Figure 9 shows the results of the original data, random sampling, SMOTE sampling, Borderline-SMOTE1 sampling, safe-level SMOTE sampling, C-SMOTE sampling, K-means SMOTE sampling and CURE-SMOTE sampling, as well as the CURE clustering result. The black circles and the red star represent the major class sample and minor class sample, respectively, in the original data, and the blue squares represent the artificial samples generated by different methods. Figure 10 shows the CURE clustering results of the minor class sample. The clustering centre is two, the stars show the centres, and the blue diamonds indicate the representative points.

Figure 9 shows that a large number of data are obtained repeatedly by random sampling, and some data are not selected at all. The SMOTE algorithm also produces repeated data and generates mixed data in other classes as well as noise. Borderline-SMOTE1 picks out the boundary point of minor class by calculating and comparing the samples of the major class around the minor class; consequently, the generated data are concentrated primarily at the edges of the class. Safe-level SMOTE follows the original distribution, but still generates repeated points and distinguishes the boundary incorrectly. Although C-SMOTE can erase the noise, the generated data are too close to the centre to accurately identify other centres. K-means-SMOTE can identify the area of the small class and slightly improves on the SMOTE effect. The proposed CURE-SMOTE algorithm generates data both near the centre and the representative points; overall, it follows the original distribution. Moreover, the representative points help to avoid noise being treated as a constraining boundary during the generating process. Detailed results are listed in Table 4.

**Table 4:** The classification results of different sampling algorithms

Dataset	Method	F	G-Mean	AUC	OOB error
1. Circle	Original data	0.9081	0.9339	0.9389	0.0296
	Random oversampling	0.9249	0.9553	0.9567	**0.0163**
	SMOTE	0.9086	0.9535	0.9579	0.0384
	Borderline-SMOTE1	0.9110	0.9534	0.9619	0.0438
	Safe-level-SMOTE	0.9146	0.9595	0.9559	0.0431
	C-SMOTE	0.9302	0.9713	0.9813	0.0702
	k-means-SMOTE	0.9262	0.9589	0.9602	0.0323
	CURE-SMOTE	**0.9431**	**0.9808**	**0.9855**	0.0323
2. Blood-transfusion	Original data	0.3509	0.5094	0.5083	0.2548
	Random oversampling	0.3903	0.5490	0.5449	0.2250
	SMOTE	0.4118	0.5798	0.5537	0.2152
	Borderline-SMOTE1	0.4185	0.5832	0.5424	**0.1630**
	Safe-level-SMOTE	0.4494	0.6174	0.5549	0.2479
	C-SMOTE	0.4006	0.5549	0.5531	0.2418
	k-means-SMOTE	0.4157	0.5941	0.5433	0.1872
	CURE-SMOTE	**0.5393**	**0.6719**	**0.6533**	0.2531
3. Haberman’s survival	Original data	0.3279	0.5018	0.6063	0.3149
	Random oversampling	0.3504	0.5178	0.5959	**0.1534**
	SMOTE	0.4350	0.5971	0.6259	0.1728
	Borderline-SMOTE1	0.4523	0.6119	0.6298	0.2589
	Safe-level-SMOTE	0.4762	0.6008	0.6030	0.3077
	C-SMOTE	0.4528	0.5487	0.5656	0.2780
	k-means-SMOTE	0.4685	0.6249	0.6328	0.1828
	CURE-SMOTE	**0.5000**	**0.6282**	**0.6940**	0.2717
4. Breast–cancer-wisconsin	Original data	0.9486	0.9619	0.9491	0.0446
	Random oversampling	0.9451	0.9623	0.9620	**0.0301**
	SMOTE	0.9502	0.9666	0.9627	0.0341
	Borderline-SMOTE1	0.9506	0.9661	0.9635	0.0379
	Safe-level-SMOTE	0.9509	**0.9671**	**0.9638**	0.0404
	C-SMOTE	0.9491	0.9636	0.9561	0.0380
	k-means-SMOTE	0.9449	0.9616	0.9562	0.0373
	CURE-SMOTE	**0.9511**	0.9664	0.9621	0.0427
5. SPECT.train	Original data	0.6348	0.6764	0.6579	0.3634
	Random oversampling	0.6539	0.6924	0.6753	0.3468
	SMOTE	0.6618	0.6990	0.6825	0.3688
	Borderline-SMOTE1	0.6710	0.6926	0.6746	0.3489
	Safe-level-SMOTE	0.6770	0.7074	0.6913	0.3160
	C-SMOTE	0.6564	0.6936	0.6764	0.3448
	k-means-SMOTE	0.6796	0.6941	0.6846	0.3599
	CURE-SMOTE	**0.6855**	**0.7155**	**0.6951**	**0.1108**

From the classification results obtained by the different sampling algorithms discussed in Table 4, the best F-value, G-mean and AUC were achieved on the Circle dataset by CURE-SMOTE, and its OOB error is second-best, behind only random sampling. The overall classification result on the blood-transfusion dataset is poorer, but the CURE-SMOTE algorithm achieves the best F-value, G-mean and AUC, while its OOB error is inferior to the original data. On the Haberman's survival dataset, the F-value, G-mean and AUC achieved by CURE-SMOTE are superior to the other sampling algorithms. For the breast-cancer-wisconsin dataset, CURE-SMOTE achieves the best F-value, but its G-mean and AUC are slightly lower, although they are little different from the other sampling algorithms. On the SPECT dataset, CURE-SMOTE surpasses the other sampling algorithms with regard to F-value, G-mean, AUC and OOB error

The best value of every performance evaluation criteria obtained by the algorithms are marked in boldface

In conclusion, the classification results of the CURE-SMOTE algorithm as measured by the F-value, G-means, and AUC are substantially enhanced, whereas the results using SMOTE alone are not particularly stable. Meanwhile, Borderline-SMOTE1, C-SMOTE, and the k-means-SMOTE algorithm are even worse than random sampling on some datasets. Thus, the CURE-SMOTE algorithm combined with RF has a substantial effect on classification.

### Experiment 2 and parameter settings

In this section, to test the effectiveness of the hybrid algorithm for feature selection and parameter optimization, we selected the representative binary classification and multi-classification imbalanced datasets shown in Table 5. These data are randomly stratified by sampling them into four parts with a training set to testing set ratio of 3:1. In this procedure, 4-fold stratified cross validation is used for classification. The parameter settings are listed in Table 6. The depth is set to 20 for experiment 2.

**Table 5:** Dataset

id	Dataset	N	M	Positive class	Negative class	IR	Label
1	Connectionist Bench	208	17	97	111	0.8739	R:M
2	Wine	130	13	59	71	0.831	1:2
3	Ionosphere	351	34	126	225	0.56	b:g
4	Breast-cancer-wisconsin	702	10	243	459	0.5249	1:0
5	Steel Plates Faults	1,941	27	**-**	-	-	7 labels
6	Libras Movement	360	90	-	-	-	15 labels
7	mfeat-factors	2,000	216	-	-	-	10 labels

**Table 6:** Parameter settings

Hybrid GA-RF	popsize :5	maxgen :20	Pc: 0.6	Pm:0.1		
Hybrid PSO-RF	popsize :5	maxgen :20	c_1_:1.5	r_1_,r_2_∈[0,1]	Vmin:Vmax = -0.5:0.5	w:0.5
			c_2_:1.5			
Hybrid AFSA-RF	popsize: 5	maxgen: 20	visual: 3	try_number: 5, delta: 0.618		

### Results and discussion of the hybrid algorithm

According to the proposed settings in previous works, the parameters for all of the methods were set as follows: *nTree* = 100, *κ* =1, $\left\lfloor \sqrt{M}\right\rfloor$, ⌊ log_2_(*M*) + 1⌋ and *M*. Accuracy, OOB error and margin were selected as the evaluation criteria. The detailed results are listed in Table 7 and Table 8. GA-RF, PSO-RF and AFSA-RF represent the hybrid algorithm.

**Table 7:** The binary classification results

		1	$\left\lfloor \sqrt{M}\right\rfloor$	⌊ log_2_(*M*) + 1⌋	*M*	GA-RF	PSO-RF	AFSA-RF
Connectionist Bench	Accuracy	0.6442	0.6442	0.6058	0.6635	0.6538	0.7308	0.6827
	Sensitive	0.5882	0.6122	0.6500	0.7556	0.5741	0.6744	0.5870
	Precision	0.6522	0.6250	0.4906	0.5862	0.7045	0.6744	0.6585
	Specificity	0.6981	0.6727	0.5781	0.5932	0.7400	0.7705	0.7586
	F	0.6186	0.6186	0.5591	0.6602	0.6327	**0.6744**	0.6207
	G-mean	0.6408	0.6418	0.6130	0.6695	0.6518	**0.7209**	0.6673
	AUC	0.4107	0.4119	0.3758	0.4482	0.4248	**0.5196**	0.4453
	OOB	0.3808	0.3889	0.3344	0.3391	0.3314	0.3085	**0.2932**
	margin	0.1078	0.1632	0.1991	0.2084	0.2056	0.1468	**0.2418**
	*nTree*	100	100	100	100	315	193	**151**
	*κ*	1	4	5	17	6	8	**4**
	*num (Attribute)*	17	17	17	17	13	16	**15**
Wine	Accuracy	0.9846	0.9692	0.9846	0.9692	0.9846	0.9846	0.9692
	Sensitive	1.0000	0.9286	1.0000	1.0000	1.0000	1.0000	1.0000
	Precision	0.9655	1.0000	0.9677	0.9333	0.9706	0.9643	0.9355
	Specificity	0.9730	1.0000	0.9714	0.9459	0.9688	0.9737	0.9444
	F	0.9825	0.9630	0.9836	0.9655	**0.9851**	0.9818	0.9667
	G-mean	0.9864	0.9636	0.9856	0.9726	0.9843	**0.9868**	0.9718
	AUC	0.9730	0.9286	0.9714	0.9459	0.9688	**0.9737**	0.9444
	OOB	0.0442	0.0502	0.0288	0.0748	0.0246	**0.0156**	0.0238
	margin	0.6951	0.7553	0.8149	0.7995	0.7863	0.7890	**0.8345**
	*nTree*	100	100	100	100	349	**354**	90
	*κ*	1	3	4	13	5	**1**	5
	*num (Attribute)*	13	13	13	13	12	**11**	12
Ionosphere	Accuracy	0.9200	0.9314	0.9371	0.9257	0.9371	0.9257	0.9314
	Sensitive	0.9107	0.8475	0.8889	0.8824	0.8333	0.9032	0.9107
	Precision	0.8500	0.9434	0.9057	0.9231	0.9804	0.8889	0.8793
	Specificity	0.9244	0.9741	0.9587	0.9533	0.9913	0.9381	0.9412
	F	0.8793	0.8929	0.8972	0.9003	**0.9009**	0.8960	0.8947
	G-mean	0.9175	0.9086	0.9231	0.9171	0.9089	0.9205	**0.9258**
	AUC	0.8956	0.8651	0.9002	0.8975	0.8548	0.8835	**0.9029**
	OOB	0.1096	0.0860	0.1132	0.0884	**0.0668**	0.0831	0.0825
	margin	0.5696	0.6918	0.6511	0.7041	**0.7349**	0.6934	0.6351
	*nTree*	100	100	100	100	**339**	321	350
	*κ*	1	5	6	34	**9**	15	2
	*num (Attribute)*	34	34	34	34	**29**	30	28
Breast -cancer -wisconsin	Accuracy	0.9801	0.9658	0.9715	0.9573	0.9544	0.9801	0.9658
	Sensitive	0.9914	0.9474	0.9583	0.9748	0.9919	1.0000	0.9474
	Precision	0.9504	0.9474	0.9583	0.9063	0.8905	0.9421	0.9474
	Specificity	0.9745	0.9747	0.9784	0.9483	0.9342	0.9705	0.9747
	F	0.9701	0.9474	0.9583	0.9393	0.9385	**0.9702**	0.9474
	G-mean	0.9829	0.9609	0.9683	0.9614	0.9626	**0.9851**	0.9609
	AUC	0.9844	0.9555	0.9595	0.9547	0.9601	**0.9850**	0.9474
	OOB	0.0422	0.0399	0.0433	0.0467	**0.0304**	0.0411	0.0372
	margin	0.8247	0.8569	0.8509	0.8652	**0.8842**	0.8179	0.8616
	*nTree*	100	100	100	100	**319**	420	351
	*κ*	1	3	4	10	**3**	1	3
	*num (Attribute)*	10	10	10	10	**9**	9	7

The best value of every performance evaluation criteria obtained by the algorithms are marked in boldface

**Table 8:** The multi-classification results

		1	$\left\lfloor \sqrt{M}\right\rfloor$	⌊ log_2_(*M*) + 1⌋	*M*	GA-RF	PSO-RF	AFSA-RF
Steel Plates Faults	Accuracy	0.7464	0.7485	0.7598	0.7814	0.7881	**0.7998**	0.7914
	OOB	0.3152	0.2819	0.2746	0.2640	0.2437	0.2276	**0.2115**
	margin	0.2456	0.3384	0.3484	0.3789	0.3803	**0.3812**	0.3810
	*nTree*	100	100	100	100	397	**283**	400
	*κ*	1	5	5	27	8	**6**	6
	*num (Attribute)*	27	27	27	27	23	**22**	22
Libras Movement	Accuracy	0.7167	0.7556	0.6889	0.6444	0.7606	0.7767	**0.7928**
	OOB	0.3546	0.3397	0.3480	0.3163	**0.3030**	0.3323	0.3116
	margin	0.1464	0.1798	0.1990	0.2180	0.2443	0.2677	**0.2910**
	*nTree*	100	100	100	100	**258**	348	135
	*κ*	1	9	7	90	**12**	8	9
	*num (Attribute)*	90	90	90	90	**56**	76	49
mfeat-fac	Accuracy	0.4280	0.9030	0.8010	0.9620	**0.9673**	0.9600	0.9611
	OOB	0.6949	0.1823	0.3192	0.0486	0.0416	0.0410	**0.0361**
	margin	−0.0987	0.4561	0.2361	0.8708	**0.8749**	0.8615	0.8698
	*nTree*	100	100	100	100	377	270	**196**
	*κ*	1	15	8	215	14	18	**11**
	*num (Attribute)*	215	215	215	215	145	112	**164**

The best value of every performance evaluation criteria obtained by the algorithms are marked in boldface

From the Connectionist Bench results, we find that the AFSA-RF achieves the minimum OOB error and the maximum margin. The best parameter combination is (151,4), and *κ* is the same as the traditional value, $\left\lfloor \sqrt{M}\right\rfloor$. The features selected by AFSA-RF were [1 1 1 1 1 1 0 1 1 0 1 1 1 1 1 1 1], meaning that the 7th and 10th features were removed. PSO-RF obtained the best F-value, G-mean and AUC. On the wine dataset, PSO-RF achieved the minimum OOB error and the maximum G-mean and AUC scores. The best parameter combination is (354,1), and *κ* is the same as the traditional value, 1. There are 15 features selected in total. Moreover, GA-RF achieved the best F-value and AFSA-RF achieved the best margin. For Ionosphere, we find that GA-RF achieved the best OOB error, F-value and margin. The best parameter combination is (339,9), but the value of *κ* is considerably different from the classic value. There are 29 total features selected. The best G-mean and AUC scores were obtained by AFSA-RF. For breast-cancer-wisconsin, we GA-RF achieved the best performance for OOB error and margin. The best parameter combination is (319,3), and *κ* is the same as the traditional value, $\left\lfloor \sqrt{M}\right\rfloor$. There are nine features selected in total. PSO-RF achieved the maximum F-value, G-mean and AUC.

The multi-classification results show that the hybrid GA-RF, PSO-RF and AFSA-RF almost always discover better features and select better parameter values than the traditional value. There, are some differences between the best *κ* and the traditional value. The more features there are originally, the greater the number of redundant features that are removed.

**Fig. 11:**
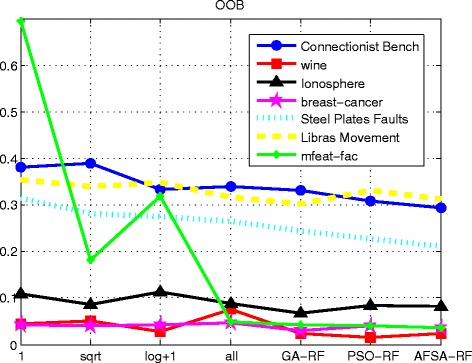
Comparison of OOB errors among different methods and datasets

Figure 11 demonstrates that, overall, the OOB error values for all the hybrid algorithms are lower than the traditional value with fixed parameters for the six datasets. Although the traditional value is reasonable for some datasets, it fails to achieve good performance over the entire problem set. In conclusion, the hybrid algorithm effectively eliminates redundant features and obtains a suitable combination of parameters. Therefore, it enhances the classification performance of RF on imbalanced high-dimensional data.

## Conclusions

To improve the performance of the random forests algorithm, the CURE-SMOTE algorithm is proposed for imbalanced data classification. The experiments show that the proposed algorithm effectively resolves the shortcomings of the original SMOTE algorithm for typical datasets and that various adaptive clustering techniques can be added to further improve the algorithm. We plan to continue to study the influence of feature selection and parameter settings on RF. The proposed hybrids of RF with intelligent algorithms are used to optimize RF for feature selection and parameter optimization. Simulation results show that the hybrid algorithms achieve the minimum OOB error, the best generalization ability and that their F-value, G-mean and AUC scores are generally better than those obtained using traditional values. The hybrid algorithm provides new effective guidance for feature selection and parameter optimization. The time and data dimensions of the experiments can be increased to further verify the algorithm’s effectiveness.

## Abbreviations

AFSA: Artificial fish swarm algorithm
AFSA-RF: Artificial fish swarm-random forests algorithm
AUC: Area under the ROC curve
Can-CSC-GBE: Cost-Sensitive Classifier with a GentleBoost Ensemble
CURE: Clustering using representatives
GA: Genetic algorithm
GA-RF: Genetic-random forests
G-mean: Geometric mean
IR: Imbalance ratio
MTD: Mega-trend-diffusion
OOB: Out of bag
PSO: Particle swarm optimization
PSO-RF: Particle swarm-random forests
RBFNN: Radial basis function neural network
RF: Random forests
ROC: Receiver operating characteristics
SMOTE: Enhances the original synthetic minority oversampling technique
SVM: Support vector machine

## References

[1] Ho TK (1995). Random decision forests [C]//Document Analysis and Recognition. Proceedings of the Third International Conference on IEEE.

[2] Ho TK (1998). The random subspace method for constructing decision forests. IEEE Trans Pattern Anal Mach Intell.

[3] Breiman L (2001). Random forests. Mach Learn.

[4] Hassan H, Badr A, Abdelhalim MB (2015). Prediction of O-glycosylation sites using random forest and GA-tuned PSO technique. Bioinform Biol Insights.

[5] Cerrada M, Zurita G, Cabrera D (2016). Fault diagnosis in spur gears based on genetic algorithm and random forest. Mech Syst Signal Process.

[6] Malik AJ, Shahzad W, Khan FA (2015). Network intrusion detection using hybrid binary PSO and random forests algorithm. Security and Communication Networks.

[7] López V, Fernández A, García S (2013). An insight into classification with imbalanced data: Empirical results and current trends on using data intrinsic characteristics. Inform Sci.

[8] Sun Y, Wong AKC, Kamel MS (2009). Classification of imbalanced data: A review. Int J Pattern Recognit Artif Intell.

[9] Khoshgoftaar TM, Golawala M, Hulse JV (2007). An empirical study of learning from imbalanced data using random forest [C]//19th IEEE International Conference on. IEEE Tools with Artificial Intelligence.

[10] Batista GE, Prati RC, Monard MC (2004). A study of the behavior of several methods for balancing machine learning training data. ACM Sigkdd Explorations Newsletter.

[11] Chen JJ, Tsai CA, Young JF (2005). Classification ensembles for imbalanced class sizes in predictive toxicology. SAR QSAR Environ Res.

[12] Pan X, Zhu L, Fan YX (2014). Predicting protein–RNA interaction amino acids using random forest based on submodularity subset selection. Comput Biol Chem.

[13] Wu Q, Ye Y, Zhang H (2014). ForesTexter: an efficient random forest algorithm for imbalanced text categorization. Knowl-Based Syst.

[14] Han M, Zhu XR (2011). Hybrid algorithm for classification of unbalanced datasets. Control Theory & Applications.

[15] Tahir M, Khan A, Majid A (2013). Subcellular localization using fluorescence imagery: Utilizing ensemble classification with diverse feature extraction strategies and data balancing. Appl Soft Comput.

[16] Ali S, Majid A, Javed SG (2016). Can-CSC-GBE: Developing Cost-sensitive Classifier with Gentleboost Ensemble for breast cancer classification using protein amino acids and imbalanced data. Comput Biol Med.

[17] Majid A, Ali S, Iqbal M (2014). Prediction of human breast and colon cancers from imbalanced data using nearest neighbor and support vector machines. Comput Methods Programs Biomed.

[18] Robnik-Sikonja M. Improving random forests [M]//Machine Learning: ECML 2004. Springer Berlin Heidelberg, 2004: 359-370.

[19] Li H B, Wang W, Ding H W, et al. Trees Weighting Random Forests Method for Classifying High-Dimensional Noisy Data [C]//2010 IEEE 7th International Conference on IEEE e-Business Engineering (ICEBE), 2010:160-163.

[20] Jian-geng L, Gao Z-k (2009). Setting of class weights in random forest for small-sample data. Comput Eng Appl.

[21] Ma J-y, Wu X-z, Xie B-c (2010). Quasi-adaptive random forest for classification. Application of Statistics and Management.

[22] Strobl C, Boulesteix AL, Kneib T, Augustin T, Zeileis A (2008). Conditional variable importance for random forests. BMC bioinformatics.

[23] Li S, James Harner E, Adjeroh DA (2011). Random KNN feature selection-a fast and stable alternative to Random Forests. BMC bioinformatics.

[24] Yang F, Lu W, Luo L (2012). Margin optimization based pruning for random forest. Neuro computing.

[25] Efron B, Tibshirani R (1993). An introduction to the boostrap [M].

[26] Breiman L (1996). Bagging predictors. Mach Learn.

[27] Quinaln J R. C4.5: programs for machine learning [M]. Morgan kuafmann, 1993.

[28] Breiman L, Friedman J, Olshen R, and Stone C. Classification and Regression Trees. Boca Raton, FL: CRC Press; 1984.

[29] He H, Garcia EA (2009). Learning from imbalanced data. IEEE Trans Knowl Data Eng.

[30] Lusa L (2010). Class prediction for high-dimensional class-imbalanced data. BMC bioinformatics.

[31] Yan H, Zha W-x (2012). Comparison on classification performance between random forests and support vector machine. Software.

[32] Chawla NV, Bowyer KW, Hall LO (2002). SMOTE: Synthetic minority over-sampling technique. J Artif Intell Res.

[33] Chawla NV, Lazarevic A, Hall LO, et al. SMOTE Boost. Improving prediction of the minority class in Boosting. In: Proceedings of the 7th European Conference on Principles and Practice of Knowledge Discovery in Databases (PKDD 2003), Lecture Notes in Computer Science, vol 2838. Springer-Verlag: Berlin; 2003. p. 107–19.

[34] Blagus R, Lusa L (2013). SMOTE for high-dimensional class-imbalanced data. BMC Bioinformatics.

[35] Han H, Wan W Y, Mao B H. Borderline-SMOTE: a new over-sampling method in imbalanced data sets learning [C]//LNCS 3644: ICIC 2005, Part I, 2005: 878-887.

[36] Bunkhumpornpat C, Sinapiromsaran K, Lursinsap C. Safe-level-SMOTE: Safe-level-synthetic minority over-sampling technique for handling the class imbalanced problem. In: Pacific-Asia Conference on Knowledge Discovery and Data Mining, Lecture Notes on Computer Science, vol 5476. Springer-Verlag: Berlin; 2009. p. 475–82.

[37] Cieslak D A, Chawla N V, Striegel A. Combating imbalance in network intrusion datasets [C]//GrC. 2006: 732-737.

[38] García V, Sánchez J S, Mollineda R A. On the use of surrounding neighbors for synthetic over-sampling of the minority class [C]//Proceedings of the 8th conference on Simulation, modeling and optimization. World Scientific and Engineering Academy and Society (WSEAS), 2008: 389-394.

[39] Peng L, Wang X-l, Yuan-chao L (2007). A classification method for imbalance data Set based on hybrid strategy. Acta Electron Sin.

[40] Zheng-feng C (2014). Study on optimization of random forests algorithm [D].

[41] Zhao W, Xu M, Jia X, et al. A Classification Method for Imbalanced Data Based on SMOTE and Fuzzy Rough Nearest Neighbor Algorithm. In: Yao Y, et al (eds) Rough Sets, Fuzzy Sets, Data Mining, and Granular Computing. Lecture Notes in Computer Science, vol 9437. Springer-Verlag: Berlin; 2015. p. 340–51.

[42] Nekooeimehr I, Lai-Yuen SK (2016). Adaptive semi-unsupervised weighted oversampling (A-SUWO) for imbalanced datasets. Expert Systems with Applications.

[43] Sáez JA, Luengo J, Stefanowski J (2015). SMOTE–IPF: Addressing the noisy and borderline examples problem in imbalanced classification by a re-sampling method with filtering. Inform Sci.

[44] Abdi L, Hashemi S (2016). To combat multi-class imbalanced problems by means of over-sampling techniques. IEEE Trans Knowl Data Eng.

[45] Guha S, Rastogi R, Shim K (1998). CURE: an efficient clustering algorithm for large databases [C]//ACM SIGMOD Record. ACM.

[46] Ya-jian Z, Xu C, Ji-guo L (2010). Unsupervised anomaly detection method based on improved CURE clustering algorithm. J Communications.

[47] Pavlidis P, Weston J, Cai J, et al. Gene functional classification from heterogeneous data. In: Proceedings of the fifth Annual International Conference on Computational Molecular Biology. 2001;249-55.

[48] Sharma A, Imoto S, Miyano S (2012). Null space based feature selection method for gene expression data. Int J Mach Learn Cybern.

[49] Ghalwash MF, Cao XH, Stojkovic I (2016). Structured feature selection using coordinate descent optimization. BMC bioinformatics.

[50] Saeys Y, Inza I, Larrañaga P (2007). A review of feature selection techniques in bioinformatics. Bioinformatics.

[51] Guo S, Guo D, Chen L (2016). A centroid-based gene selection method for microarray data classification. J Theor Biol.

[52] Sharbaf FV, Mosafer S, Moattar MH (2016). A hybrid gene selection approach for microarray data classification using cellular learning automata and ant colony optimization. Genomics.

[53] Golub TR, Slonim DK, Tamayo P (1999). Molecular classification of cancer: class discovery and class prediction by gene expression monitoring. Science.

[54] Furey TS, Cristianini N, Duffy N (2000). Support vector machine classification and validation of cancer tissue samples using microarray expression data. Bioinformatics.

[55] Sharma A, Imoto S, Miyano S (2012). A top-r feature selection algorithm for microarray gene expression data. IEEE/ACM Transactions on Computational Biology and Bioinformatics (TCBB).

[56] Chinnaswamy A, Srinivasan R. Hybrid Feature Selection Using Correlation Coefficient and Particle Swarm Optimization on Microarray Gene Expression Data. In: Snášel V, et al (eds) Innovations in Bio-Inspired Computing and Applications. Advances in Intelligent Systems and Computing, vol 424. Springer International Publishing Switzerland; 2016. p. 229-39.

[57] Destrero A, Mosci S, De Mol C (2009). Feature selection for high-dimensional data. Comput Manag Sci.

[58] Zhu S, Wang D, Yu K (2010). Feature selection for gene expression using model-based entropy. IEEE/ACM Trans Comput Biol Bioinform.

[59] Kausar N, Majid A (2016). Random forest-based scheme using feature and decision levels information for multi-focus image fusion. Pattern Anal Applic.

[60] Menze BH (2009). A comparison of random forest and its Gini importance with standard chemometric methods for the feature selection and classification of spectral data. BMC bioinformatics.

[61] Strobl C (2007). Bias in random forest variable importance measures: Illustrations, sources and a solution. BMC bioinformatics.

[62] Zhou Q, Zhou H, Li T (2016). Cost-sensitive feature selection using random forest: Selecting low-cost subsets of informative features. Knowl-Based Syst.

[63] Díaz-Uriarte R, De Andres SA (2006). Gene selection and classification of microarray data using random forest. BMC bioinformatics.

[64] Lariviere B, Van den Poel D (2005). Predicting customer retention and profitability by using random forests and regression forests techniques. Expert Systems with Applications.

[65] Rodriguez-Galiano VF, Ghimire B, Rogan J, Chica-Olmo M, Rigol-Sanchez JP (2012). An assessment of the effectiveness of a random forest classifier for landcover classification. ISPRS J Photogramm Remote Sens.

[66] Bernard S, Heutte L, Adam S (2009). Influence of Hyper parameters on Random Forest Accuracy [C]//Proceedings of the 8th International workshop on multiple classifier systems.

[67] Yu L, Chun-xia Z (2011). Estimation of the hyper-parameter in random forest based on out-of-bag sample. J Syst Eng.

[68] Martinez-Munoz G, Suarez A (2010). Out-of-bag estimation of the optimal sample size in bagging. Pattern Recogn.

[69] Ming-yuan Z, Yong T, Chong F, Ming-tian Z (2010). Feature selection and parameter optimization for SVM based on genetic algorithm with feature chromosomes. Control and Decision.

[70] Lei L, Gao L, Shijie Z (2015). Question of SVM kernel parameter optimization with particle swarm algorithm based on neural network. Comput Eng Appl.

[71] Leifu GAO, Shijie ZHAO, Jing GAO (2013). Application of artificial fish-swarm algorithm in SVM parameter optimization selection. Comput Eng Appl.

[72] Xin-guang SHAO, Hui-zhong YANG, Gang CHEN (2006). Parameters selection and application of support vector machines based on particle swarm optimization algorithm. Control Theory & Applications.

[73] Kennedy J,Eberhart R. Particle Swarm Optimization [C]//IEEE International Conference on Neural Networks,1995 Proceedings,1995:1942–1948.

[74] Xiao-lei L, Zhi-jiang S, Ji-xin Q (2002). An optimizing method based on autonomous animals: Fish-swarm Algorithm. Systems Engineering-Theory & Practice.

[75] Chen J, Tang YY, Fang B (2012). In silico prediction of toxic action mechanisms of phenols for imbalanced data with Random Forest learner. J Mol Graph Model.

[76] Espíndola R P, Ebecken N F F. On extending f-measure and g-mean metrics to multi-class problems [C]//Sixth international conference on data mining, text mining and their business applications, Wessex Institute of Technology, UK. 2005, 35: 25-34.

[77] Janitza S, Strobl C, Boulesteix AL (2013). An AUC-based permutation variable importance measure for random forests. BMC bioinformatics.

[78] Breiman L (1996). Out-of-bag Estimation [R].

[79] Mellor A, Boukir S, Haywood A (2015). Exploring issues of training data imbalance and mislabeling on random forest performance for large area land cover classification using the ensemble margin. ISPRS J Photogramm Remote Sens.

